# Prevalence of risky sexual behavior and associated factors among Injibara University students, Northwest Ethiopia

**DOI:** 10.3389/frph.2024.1356790

**Published:** 2024-03-28

**Authors:** Mekdes Abera Adal, Saron Abeje Abiy, Mebratu Mitiku Reta, Mezgebu Silamsaw Asres, Yaregal Animut

**Affiliations:** ^1^ Injibara District Health Office, Awi Zone Health Department, Amhara Regional Health Bureau, Injibara, Ethiopia; ^2^ Department of Clinical Midwifery, School of Midwifery, College of Medicine and Health Sciences, University of Gondar, Gondar, Ethiopia; ^3^ Department of Internal Medicine, School of Medicine, College of Medicine and Health Sciences, University of Gondar, Gondar, Ethiopia; ^4^ Department of Epidemiology and Biostatistics, Institute of Public Health, College of Medicine and Health Sciences, University of Gondar, Gondar, Ethiopia

**Keywords:** prevalence, risky sexual behavior, associated factors, university students, Northwest Ethiopia

## Abstract

**Introduction:**

Engaging in risky sexual behaviors can lead to HIV infection, sexually transmitted infections, and unintended pregnancy among youths. University students had greater sexual risks for many reasons. Therefore, this study aimed to assess the magnitude and associated factors of risky sexual behaviors among regular undergraduate students at Injibara University, Northwest Ethiopia.

**Methods:**

A cross-sectional study was conducted at Injibara University from 20 January to 30 2020. Multistage sampling was employed to select 770 students. Data were collected using a semistructured self-administered questionnaire. A binary logistic regression model was used to identify factors associated with risky sexual behavior. Adjusted odds ratios with 95% CIs were determined, and variables with *P*-values <0.05 were considered significant.

**Result:**

A total of 770 students participated in the study, providing a response rate of 100%. In this study, 294 (38%, 95% CI: 35%, 42%) students engaged in risky sexual behaviors. Risky sexual behavior was significantly associated with not tested for HIV (AOR = 1.62, 95% CI: 1.15–2.31), peer pressure (AOR = 1.90, CI: 1.37–2.64), basic HIV knowledge (AOR = 2.16, CI: 1.65–2.89), substance use (AOR = 3.56, CI: 2.11–6.06), watching pornography videos (AOR = 1.58, CI: 1.11–2.23), and HIV risk perception (AOR = 1.37, CI: 1.02–1.91).

**Conclusion and recommendation:**

A substantial proportion of university students in this study engaged in unsafe sexual behavior. Risky sexual behaviors are more likely to occur when students are under peer pressure, use substances, have no perceived HIV risk, watch pornography, and have inadequate basic HIV knowledge. Therefore, tailored strategic interventions such as life skill training should be designed to bring about positive behavioral changes among university students.

## Introduction

Risky sexual behavior is defined as any sexual activity that increases the risk of contracting sexually transmitted infections (STIs) and unintended pregnancy, which includes having sex with multiple sexual partners, early initiation of sexual intercourse under the age of 18 years, and unprotected sex (1, 2).

Sexual and reproductive health problems are among the main causes of death, disability, and disease among young people worldwide. According to the Centers for Disease Control and Prevention (CDC) report, 20% of all new HIV diagnoses and more than half of the nearly 20 million new STIs were among young people (aged 15–24 years) in 2020, and more than 145,000 infants were born to adolescent females in 2021 (1). HIV/AIDS is the second leading cause of death globally and the leading cause of death in Africa among young people (2). Globally, approximately 14,000 new HIV infections occur daily due to risky sexual behavior, with more than 95% of them in developing countries. Particularly, in sub-Saharan Africa, HIV/AIDS and unintended pregnancies continue to be major causes of mortality and morbidity among adolescents (3–5). According to the Ethiopian Demographic and Health Survey (EDHS) report, the percentage of first sexual intercourse among young people in Ethiopia aged between 18 and 24 who have had sex before age 18 has increased from 35% in 2016 to 40% in 2019 among women and from 9% to 12% among men (6, 7).

The transition from high school to university on its own has been identified as a major life stressor for many young people (8). During this period, many students live away from their homes for the first time, which may result in a great decrease in contact and support from family and friends (9). In addition, in this period, students have to deal with greater autonomy, enhanced academic demands and new social relationships. Furthermore, most university youths take risks due to a lack of knowledge, or they think that they are invulnerable (10). During the process of dealing with these university life challenges, students may engage in risky sexual behaviors that cause poor health outcomes both physically and mentally (11).

In Ethiopia, sexual matters are very sensitive and less likely to be discussed openly. Little attention is given to sexual and reproductive services for youths (12). Although youths are assets of society and change agents in filling the gap in the past and on whom the future generation is based, most HIV-related interventions target the general public; as a result, they do not directly respond to higher education students' needs, making actual coverage of behavioral and biomedical interventions for youths extremely low (13). Neglecting their sexual and reproductive health can lead to high social and economic costs, both immediately and in the years ahead (13). Understanding the determinants of university youth's risky sexual behaviors is an essential step toward curtailing the spread of STIs/HIV/AIDS and unintended pregnancies. Therefore, this study aimed to assess the magnitude and factors associated with risky sexual behavior among undergraduate regular students at Injibara University.

## Methods

### Study design and period

An institution-based cross-sectional study was conducted among regular students at Injibara University from January 20 to 30, 2020 G.C.

### Study area and setting

The study was conducted at Injibara University located in the Awi zone of the Amhara region, 114 km from Bahir-Dar and 447 km from Addis Ababa, the nation's capital. According to the data from the registrar and alumni directorate, there were 2,269 regular undergraduate students (1,282 males and 1,024 females) in the university at the beginning of the 2019/20 academic year. Of the total regular students, 1,245 were in their second year, and 1,024 were in their third year. First-year students for the 2019/20 academic year were not registered at the time of data collection.

### Study population

All Injibara University undergraduate regular students during the study period were included in this study.

Inclusion criteria:

All Injibara University undergraduate regular students during the study period were included in this study regardless of having or not having their first sexual intercourse. Exclusion criteria:

Students with vision disability were excluded from the study as they can't fill self-administered questionnaire.

### Sample size and sampling technique

The sample size was determined using a single population proportion formula using the following assumptions: the probability of an event from the previous study was 35%, the proportion of students who had experienced risky sexual behaviors in a study conducted at Mizan Aman College of Health Sciences (14), the probability of type 1 error or alpha was 0.05, and the 95% confidence level. The sample size was also calculated using the double population proportion formula for the second objective by considering different odds ratios (ORs) of significant factors from previous studies, including alcohol consumption, peer pressure and khat chewing. However, the largest sample size, which was found in the following formula, was considered as the final sample size.

$$n\;=\;[(Za/2{)}^{2}p\;(1-p)]/d^{2}\;=\;350,$$

Adding a 10% nonresponse rate and design effect 2, we use a final sample size of 770.

A multistage sampling technique was employed to select students from four colleges of Injibara University (Social Sciences and Humanities College, Business and Economics College, Agriculture, Food and Climate College, and Natural and Computational Sciences College). Samples were proportionally allocated to each college and then further allotted based on the year of study and the number of males and females proportionally. Finally, participants were selected randomly using the lottery method from the selected class.

### Variables of the study

**Dependent Variables**: Risky sexual behavior (Yes/No).

### Independent variables

**Sociodemographic variables**: Age, sex, religion, parental education level, pocket money, marital status, and year of study.

**Knowledge and psychosocial factors**: HIV risk perception, use of VCT, peer pressure, knowledge of contraceptive methods, and basic HIV knowledge.

**Behavioral factors**: Substance use, alcohol, khat, cigarette and social media use pornography videos, Facebook.

### Operational definition

**Risky sexual behaviors**: a student who practiced at least one of the following**:** early initiation of sexual intercourse, unprotected sex, having sex with sex workers, having sexual intercourse with multiple sexual partners and practicing sexual intercourse while under the influence of substances in the last 12 months (14–18).

**Early initiation of sex**: a student who experienced sexual intercourse before 18 years old (14, 19).

**Unprotected sex**: a student who did not use a condom at every act of sexual intercourse (sex without a condom or inconsistent use of a condom) was considered to have unprotected sex.

**Multiple Sexual Partners**: a student who had more than one sexual partner in the last 12 months prior to the study (14).

**Sexually active**: a student who reported that she/he has had sexual intercourse, irrespective of their marital status at least once prior to the study (20).

**Basic HIV knowledge:** A participant was considered to have basic knowledge of HIV transmission and prevention methods if he/she responded “YES” to the following two questions: (1) People can reduce their chance of getting HIV by having just one uninfected and faithful sexual partner, and (2) People can reduce their chance of getting HIV by using a condom every time they have sex (6).

**HIV Risk Perception**: Students were asked “What are the chances that you might get HIV?” Responses include no chance, low chance, moderate chance, high chance, and do not know. Then, the data were dichotomized into “Yes” and “No” (21).

**Knowledge of contraceptive methods**: a student who could list at least one modern contraceptive method was considered knowledgeable (22).

**Substance use**: a student who has consumed at least one of the substances (khat, cigarettes, and illicit drugs) in any amount in the last 12 months is considered a substance user (23, 24).

### Data collection tools and procedure

A pretested and semi structured Amharic-version self-administered questionnaire was used to collect the data. The questionnaire was first prepared in English and then translated to Amharic and back to English by language experts to check consistency and conceptual similarity. Two MSc instructor supervisors and four BSc data collectors were recruited to collect the data. The data collectors and supervisors were trained for 1 day to make them familiar with the questionnaires and the way how to orient the study participants in filling the questionnaire. A pretest was conducted on 5% of the sample size at the University of Gondar before the actual data collection period, and the necessary revisions of questions were made.

Questionnaires were distributed among randomly selected students just after university lessons, and the filled questionnaires were collected on the same day. The selected students were seated at a reasonable distance when they filled out the questionnaire, and they were provided with a box with an opening at the front desk into which they placed all completed questionnaires to ensure anonymity and also to reduce social desirability bias The data collection process was closely monitored by the principal investigator and the supervisors throughout the data collection period. Filled questionnaires were checked regularly for completeness of information, and any problems were immediately discussed with the data collectors.

### Data processing and analysis

Data were entered using Epi Info version 7 and exported to SPSS version 25 for analysis. Frequency tables, percentages, and means were used to describe the data. Risky sexual behavior was coded as “1” = “Yes” and “0” = “No” then univariate binary logistic regression was fitted to screen factors for the multivariable binary logistic regression. The Chi-square (*χ*^2^) assumption was checked for each variable before fitting the univariate binary logistic regression analysis. Variables with *P*-values ≤0.2 in the bivariable analysis were entered into the multivariable analysis to identify independent factors associated with risky sexual behavior. The adjusted odds ratio (AOR) with 95% CI was calculated, and statistical significance was declared at a *P*-value of 0.05 in the multivariable analysis. The goodness of fitness of the model was checked using Hosmer and Lemeshow test.

## Results

### Sociodemographic characteristics

A total of 770 students participated in the study, providing a response rate of 100%. The mean age of the participants was 23.02 (±2.52 SD) years, and the majority of the participants (586, 76.1%) were between the age groups of 18 and 24 years. More than half 397 (51.6%) of the participants were males, and only 30 (3.9%) were ever married. More than three-fourths 599 (77.8%) of the respondents were Orthodox Christian followers, and nearly two-thirds 482 (62.6%) of the participants were second-year students (Table 1).

**Table 1:** Sociodemographic characteristics of regular undergraduate students at Injibara University, Northwest Ethiopia, January 2020 (*n* = 770).

Variables	Categories	Frequency	Percent
Sex	Male	397	51.6
	Female	373	48.4
Age in years	18–24	586	76.1
	≥25	184	23.9
Marital status	Single	745	96.75
	Married	25	3.25
Religion	Orthodox	599	77.8
	Muslim	81	10.5
	Protestant	68	8.8
	Others*	22	2.9
Mother educational level	Unable to read and write	203	26.4
	Read and write only	234	30.4
	Primary school	150	19.5
	secondary school	112	14.5
	Collège and above	71	9.5
Father educational level	Unable to read and write	169	21.9
	Read and write only	252	32.7
	Primary school	203	26.4
	Secondary school	68	8.8
	Collège and above	78	10.1
Year of study	Second year	482	62.6
	Third year	288	37.4
Monthly pocket money	≤500ETB	613	79.61
	>500ETB	157	20.39

* Others  = Catholic, traditional.

### Substance and social media use

More than one in ten students 93 (12.08%) were substance users. Of these, 68 (8.8%) chewed khat, 57 (7.4%) smoked cigarettes, and 40 (5.2%) smoked Hashish. Nearly two-thirds of the participants [484 (62.9)] used Facebook, and 246 (31.9%) had a habit of watching pornography (Table 2).

**Table 2:** Substance and social media use among regular undergraduate students at Injibara University, Northwest Ethiopia, January 2020 (*n* = 770).

Variables	Frequency	Percent
Alcohol drinking		
Yes	240	31.2
No	530	68.8
Khat chewing		
Yes	68	8.8
No	702	91.2
Cigarette smoking		
Yes	57	7.4
No	713	92.6
Hashish use		
Yes	40	5.2
No	730	94.8
Facebook use		
Yes	484	62.9
No	286	37.1
Watch pornography		
Yes	246	31.9
No	524	68.1

### Sexual behaviors and HIV-related knowledge

Of the total respondents, 470 (61%) had basic knowledge of HIV transmission and prevention. More than one-third (281, 36.5%) of the participants perceived that they were not at risk of HIV infection. More than two-thirds (542, 70.4%) of the students ever tested for HIV. Nearly half 346 (45%) of the respondents were sexually active, of whom 208 (60%) were males and 138 (40%) were females. The mean age at first sexual intercourse was 18.01 years (SD ± 2.35 years) (18.56 years (SD ± 2.1 years) for males and 17.18 years (SD ± 2.5 years) for females (Table 3).

**Table 3:** Sexual behaviors and HIV-related knowledge among regular undergraduate students at Injibara University, Northwest Ethiopia, January 2020.

Variables	Categories	Frequency	Percent
Basic HIV transmission and prevention knowledge	Yes	470	61
	No	300	39
HIV risk perception	Yes	489	63.5
	No	281	36.5
Ever tested for HIV	Yes	542	70.4
	No	228	29.6
Peer pressure for sex	Yes	367	47.7
	No	403	52.3
Ever had sexual intercourse	Yes	346	44.9
	No	424	55.1
Age at first sexual intercourse (*n* = 346)	<18 years	131	37.9
	≥18 years	215	62.1
Life time number of sexual partners (*n* = 346)	1	246	71.1
	≥2	100	28.9
Number of sexual partners in the last one year (*n* = 346)	none	99	28.6
	1	169	48.8
	≥2	78	22.5
History of sexual intercourse with FSW (for males) (*n* = 208)	Yes	75	36.1
	No	133	63.9
Sex while under the influence of substance (*n* = 346)	Yes	77	22.3
	No	269	77.7
Condom use (*n* = 346)	Inconsistent	185	53.5
	Consistent	107	30.9
	Never used	54	15.6
Condom use during the last sex (*n* = 346)	Yes	153	44.2
	No	193	57.8

**Figure 1:**
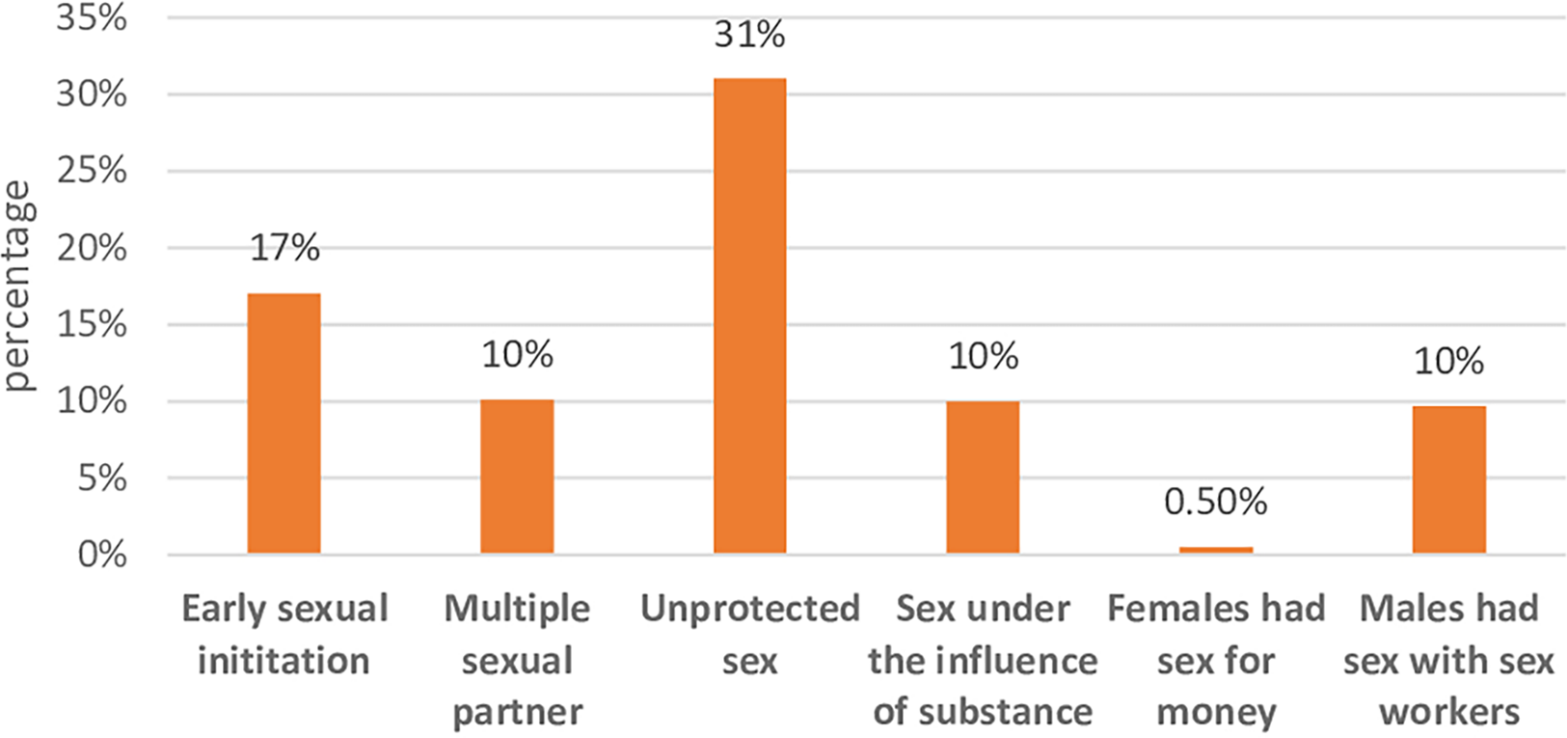
Risky sexual behaviors among Injibara undergraduate regular University students, Northwest Ethiopia, January, 2020 (*n* = 770).

More than one-third 294 (38.2%) of the participants had risky sexual behavior. Of these, 131 (17%) of the participants started sex before the age of 18 years, 78 (10%) had more than one sexual partner in the last year, 77 (10%) had sexual intercourse under the influence of substance, and 239 (31%) did not use condoms consistently (Figure 1).

More than half 193 (57.8) of the respondents did not use condoms during their last sexual intercourse. Among sexually active females, 4 (2.9%) had sex for money, and more than one-third of 75 (36.1%) sexually active males had sexual intercourse with female sex workers.

Of the total respondents, 274 (73.5%) females and 222 (56%) males had knowledge of contraceptive methods. Nearly two-thirds (88, 63.8%) of sexually active females had used at least one type of modern contraceptive method, and 16 (11.6%) females had a history of unwanted pregnancy or abortion.

### Factors associated with risky sexual behaviors

In this study, factors such as, tested for HIV, basic HIV knowledge, HIV risk perception, peer pressure, watching pornography films, and substance use were associated with risky sexual behavior in the multivariable analysis.

Students who had never been tested for HIV were 1.62 times (AOR = 1.62, 95% CI: 1.15–2.31) more likely to have risky sexual behavior than those who had been tested for HIV. Similarly, students who lacked basic knowledge of HIV were 2.16 times (AOR = 2.16, 95% CI: 1.56–2.98) more likely to engage in RSB than those who had basic HIV knowledge.

Participants who faced peer pressure for sexual intercourse were approximately 2 times (AOR = 1.90, 95% CI: 1.37–2.64) more likely to have risky sexual behavior than those who did not face peer pressure.

Participants who use substances were 3.56 times (AOR = 3.56, 95% CI: 2.11–6.06) more likely to have risky sexual behavior than those who did not use substances. Participants who watched pornography films were 1.58 times (AOR = 1.58, CI: 1.11–2.23) more likely to be involved in risky sexual behavior than those who did not watch pornography films. Students who had no perceived HIV risk were 1.37 times (AOR = 1.37, CI: 1.02–1.19) more likely to have risky sexual behavior than those who had HIV risk perception (Table 4).

**Table 4:** Factors associated with risky sexual behaviors among Injibara University undergraduate regular students, Northwest Ethiopia, 2020 (*n* = 770).

Variables	Categories	Risky sexual behaviours		COR (95% CI)	AOR (95% CI)	*P*-value
		Yes	No			
Marital status	Single	290	455	3.35 (1.14, 9.85)*	2.58 (0.84, 7.89)	0.096
	Married	4	21	1	1	
Study year	2nd year	195	287	1.30 (0.96, 1.76)	1.41 (0.98, 1.97)	0.15
	3rd year	99	189	1	1	
HIV tested	Yes	189	353	1	1	
	No	105	123	1.59 (1.16, 2.18)*	**1.62** (1.15, 2.31)*	**0**.**006**
Basic HIV knowledge	Yes	148	322	1	1	** **
	No	146	154	2.06 (1.53 (2.78)**	**2.16** (1.56, 2.98)**	**0 < 0**.**001**
Contraceptive knowledge	Yes	213	286	1.75 (1.28, 2.39)*	1.48 (0.98,2.11)	0.26
	No	81	190	1	1	
Substance use	Yes	64	29	4.29 (2.69, 6.84)**	**3.56** (2.11, 6.06)**	**0 < 0**.**001**
	No	230	447	1	1	** **
Peer pressure	Yes	170	197	1.94 (1.45, 2.61)**	**1.90** (**1.37, 2.64)**	**0 < 0**.**001**
	No	124	279	1	1	** **
Watch pornography	Yes	127	119	2.28 (1.67, 3.11)**	**1.58** (**1.11, 2.23)**	**0**.**01**
	No	167	357	1	** **	1
Alcohol drinking	Yes	112	128	1.67 (1.23, 2.28)	1.01 (0.70, 1.45)	0.97
	No	182	348	1	1	
HIV risk perception	Yes	177	312	1	1	** **
	No	117	164	1.26 (0.93, 1.70)	**1.37** (1.02, 1.91)*	**0**.**05**

## Discussion

In this study, the prevalence of risky sexual behavior was 38.2% (95% CI: 35%–42%). This finding was comparable with studies conducted in governmental higher institution students of Debre-Markos town 42% (25), Jimma University 42.1% (26), Mizan Aman University 35% (18), and a systematic review and meta-analysis findings of risky sexual behavior among students in Ethiopian universities 42.8% (17) and 41.62% (27). However, this finding is higher than studies conducted in Arbaminch University students, where the prevalence was 31.4% (28), Aksum University 17% (29), Wolkitie University 20% (30), Debre-Tabor University 28.4% (31), Sri Lanka 12.4% (32), and Southern Nigeria 17.9% (33).

On the other hand, the prevalence of risky sexual behavior in our study is lower than that in studies conducted at Debre-Markos University (58.2%) (34), Jig-Jiga University (54.8%) (35), Bahir-Dar University (62%) (36), Medawalabu University (70.9%) (37), South Africa (81%) (38), Turkey (51.9%) (39), and Brazil (97%) (40). This difference might be due to approaches taken to quantify risky sexual behavior and sociocultural differences. For example, in our study, we assessed multiple sexual partners 12 months prior to the study, while others considered having had more than one sexual partner any time in the past. On the other hand, the study from Bahir-Dar considered any student who is sexually active as having risky sexual behavior (36).

The study revealed that respondents who had never been tested for HIV were 1.62 times more likely to have risky sexual behaviors than those who had been tested for HIV. This finding is supported by evidence from Malawi (41) and the USA (42). The possible reason might be that risk reduction counseling by health care workers to those who tested could help to reduce risky sexual behavior (43).

This study showed that participants who lack basic knowledge of HIV/AIDS transmission and prevention methods are two times more likely to engage in risky sexual behaviors than their counterparts. This finding is supported by studies conducted in Nigeria (44), China (45), and the USA (46). The reason could be that participants who have good knowledge will outweigh the risk of contracting HIV and will be cautious in their sexual relationships (47, 48).

In this study, respondents who consume substances such as smoking cigarette or chewing chat or consume hashish were approximately 3.5 times more likely to practice risky sexual behaviors than their counterparts. This was again supported by studies conducted among students at Madawalabu University (37), Bahir-Dar University (36), Debre Markos University (34), Uganda (49), Debre-Tabor University (31), and studies in nine Asian Countries (50). Usually, substance use and risky sexual behavior go hand in hand (51). This might be due to the nature of these substances, which alter rational decision-making ability, decrease inhibitions and increase the risk-taking behavior of individuals, leading them to have unsafe sex (52).

Peer pressure showed a significant association with risky sexual behavior, with a double increase in the odds of RSB among students with peer pressure compared to students with no peer pressure. This finding was supported by studies conducted among students in Jimma University (53), Nigeria (54), Rwanda (55), and Adama Ethiopia (56). This might be because students share their day-to-day life experience with their friends, and they are likely to behave in a manner intimate friends practice (57). Evidence has revealed that peers have significant influences on young people's behavior (58). Strengthening peer education programs is essential to fostering a culture of healthy peer influence.

Respondents who watched pornography films were more likely to have risky sexual behavior compared to their counterparts. Studies at Bahir-Dar University (36) and a meta-analysis finding among college and university students in Ethiopia (27) supported our finding. The reason might be that watching pornography increases the motivation of sexual desire. Furthermore, adolescent students are sensitive to experimenting with what they hear and look as a result of the natural transition stage to adulthood; hence, they are prone to be driven by porn videos they watch to experiment with risky sex (2, 59).

Participants who had no perceived HIV risk were more likely to have risky sexual behavior than those who had perceived HIV risk. This meant that those who thought themselves not at risk of infection to HIV were more likely to practice risky sexual behavior, which might be associated with underestimating the risk of acquiring HIV infection (60, 61). However, this finding was contrary to findings from studies conducted at Mizan-Aman University (14) and Zimbabwe University (62). This implies that students perceive themselves to be at risk of HIV infection considering their involvement in risky sexual behavior (63, 64).

Based on our findings, it is essential to consider strategic interventions designed to improve the sexual and reproductive health of University students. Implementing sexual educational programs to increase awareness about HIV transmission, prevention methods, and safe sexual practices could be important (61, 65, 66). This could be achieved through peer education programs (67), incorporating sexual health education into the university curriculum across various disciplines (68–71), and through social media where adolescents will be interested in reproductive and sexual health messages (72, 73). Offering easy access to and encouraging confidential HIV testing services on campus, accompanied by counseling and support services, is crucial (74). Making condoms and other reproductive health services readily available and accessible in campus health centers, dormitories, and other relevant locations is also essential (75, 76). Implementing tailored interventions for high-risk groups among university students, such as substance users, female students, and those engaging in unprotected sex, is vital (77–79).

## Limitations of the study

Our study could be affected by social desirability bias. Therefore, the magnitude of risky sexual behavior could be underestimated due to the sensitive nature of the topic. Sexuality and sex-related topics are particularly more sensitive among adolescents due to the societal outlook and attitude toward sex at a young age. However, this could be minimized since we used a self-administered questionnaire to collect the data. In addition, in this study, we could not establish a temporal relationship between the factors and the outcome due to the cross-sectional nature of the study.

The study was limited to university students, so the results may not be generalizable to other adolescents with diverse lifestyles. Thus, the findings of this study should be interpreted with these limitations.

## Conclusion

The prevalence of risky sexual behavior among undergraduate regular university students was high. Risky sexual behaviors are more likely to occur when students are under peer pressure, use substances, have no perceived HIV risk, watch pornography, and have inadequate basic HIV knowledge. Therefore, strategies such as life skill training should be designed to bring positive behavioral changes among university students.

## Data Availability

The original contributions presented in the study are included in the article/Supplementary Material, further inquiries can be directed to the corresponding author.

## References

[1] Centers for Communicable Diseases Control and Prevention (CDC). Adolescent and school health, sexual risk behaviors (2021). Available online at: https://www.cdc.gov/healthyyouth/sexualbehaviors/index.htm (accessed January 16, 2022).

[2] UNAIDS. Active Involvement of Young People Agains HIV/AIDS. New York: UNAIDS (2015).

[3] World Health Organization. Sexual health and its linkages to reproductive health: an operational approach (2017).

[4] Yakubu I Salisu WJ. Determinants of adolescent pregnancy in sub-Saharan Africa: a systematic review. Reprod Health. (2018) 15(1):15. 10.1186/s12978-018-0460-4 29374479 PMC5787272

[5] Idele P Gillespie A Porth T Suzuki C Mahy M Kasedde S Epidemiology of HIV and AIDS among adolescents: current status, inequities, and data gaps. JAIDS J Acquir Immune Defic Syndr. (2014) 66:S144–53. 10.1097/QAI.0000000000000176 24918590

[6] Central Statistical Agency (CSA) [Ethiopia] and ICF. Ethiopia Demographic and Health Survey 2016. Addis Ababa, Ethiopia, and Rockville, Maryland, USA: CSA and ICF (2017).

[7] Ethiopian Public Health Institute (EPHI) [Ethiopia] and ICF. Ethiopia Mini Demographic and Health Survey 2019: Final Report. Rockville, Maryland, USA: EPHI and ICF (2021).

[8] Bray SR Born HA. Transition to university and vigorous physical activity: implications for health and psychological well-being. J Am Coll Health. (2004) 52(4):181–8. 10.3200/JACH.52.4.181-188 15018429

[9] Tajalli P Sobhi A Ganbaripanah A. The relationship between daily hassles and social support on mental health of university students. Procedia Soc Behav Sci. (2010) 5:99–103. 10.1016/j.sbspro.2010.07.058

[10] Simons-Morton B. Reducing Adolescent Risk: Toward an Integrated Approach. 2nd ed. New York, USA: SAGE Publications, Inc (2012). p. 536.

[11] Jackson ES Tucker CM Herman KC. Health value, perceived social support, and health self-efficacy as factors in a health-promoting lifestyle. J Am Coll Health. (2007) 56(1):69–74. 10.3200/JACH.56.1.69-74 17711829

[12] Kabbash IA El Sayed NM Al Nawawy AN Shady IK Abou Zeid MS. Condom use among males 15–49 years aged in lower Egypt. East Mediterr Health J. (2007) 13(6):1405–17. 10.26719/2007.13.6.1405 18341190

[13] WHO. Investing in Our Future: A Framework for Accelerating Action for the Sexual and Reproductive Health of the Young People. Geneva: WHO (2006). p. 2–6.

[14] Yarinbab TE Tawi NY Darkiab I Debele F Ambo WA. Risky sexual behaviors and associated factors among students of mizan aman college of health science, Southwest Ethiopia: cross-sectional study. JOJ Nurs Health Care. (2018) 8(3):805–12. 10.19080/JOJNHC.2018.08.555736

[15] Tadesse GM. Determinants of Adolescent Risky Sexual Behavior and Possible Effective Interventions in Ethiopia. Vrije Universiteity: Amsterdam (2015).

[16] Karen S. Substance use and sexual risk taking in adolescence. Act Youth Center Excellence. (2012) 9:1–9. https://actforyouth.net/resources/rf/rf_substance_0712.pdf

[17] Muche AA Kassa GM Berhe AK Fekadu GA. Prevalence and determinants of risky sexual practice in Ethiopia: systematic review and meta-analysis. BMC. (2017) 14(113). 10.1186/s12978-017-0376-4 PMC5588747 28877736

[18] Kebede A Molla B Gerensea H. Assessment of risky sexual behavior and practice among Aksum University students, Shire Campus, Shire Town, Tigray, Ethiopia, 2017. BMC Res Notes. (2018) 11(1):88. 10.1186/s13104-018-3199-7 29386042 PMC5793377

[19] Kassahun EA Gelagay AA Muche AA Dessie AA Kassie BA. Factors associated with early sexual initiation among preparatory and high school youths in Woldia town, Northeast Ethiopia: a cross-sectional study. BMC Public Health. (2019) 19(1):378. 10.1186/s12889-019-6682-8 30947690 PMC6450012

[20] Olika AK Kitila SB Terfa YB Olika AK. Contraceptive use among sexually active female adolescents in Ethiopia: trends and determinants from national demographic and health surveys. Reprod Health. (2021) 18(1):104. 10.1186/s12978-021-01161-4 34034741 PMC8146240

[21] Haile Z Kingori C Darlington K-A Basta T Chavan B. HIV risk perception among college students at a university in the midwest. Sex Cult. (2017) 21(1):62–73. 10.1007/s12119-016-9380-z

[22] Nsubuga H Sekandi JN Sempeera H Makumbi FE. Contraceptive use, knowledge, attitude, perceptions and sexual behavior among female university students in Uganda: a cross-sectional survey. BMC Women’s Health. (2016) 16(1):6. 10.1186/s12905-016-0286-6 26818946 PMC4730721

[23] Shegute T Wasihun Y. Prevalence of Substance Use in University Students, Ethiopia. J Subst Abuse Treat. (2021) 15. 10.1177/11782218211003558 PMC8013928 33854324

[24] Tesfaye G Derese A Hambisa MT. Substance use and associated factors among university students in Ethiopia: a cross-sectional study. J Addict. (2014) 2014:1–8. 10.1155/2014/969837 PMC4020400 24872903

[25] Mekonnen M Yimer B Wolde A. Sexual risk behaviour and associated factors among governmental higher institution students in Debre Markos town. Public H Open Acc. (2018) 2(1):3–5. 10.233880/phoa-16000121

[26] Abdu S Tesfaye HB FeKecha HB. Assessment of risky sexual behaviour and associated factors among Jimma university students. Prim Health Care. (2017) 7(2):1–4. 10.4172/2167-1079.1000268

[27] Amare T Yeneabat T Amare Y. A systematic review and meta-analysis of epidemiology of risky sexual behaviors in college and university students in Ethiopia, 2018. J Environ Public Health. (2019) 2019:4852130. 10.1155/2019/4852130 PMC6446110 31015844

[28] Soboka B Kejela G. Assessment of risky sexual behaviors among arba minch university students, arba minch town, snnpr, Ethiopia. J Child Adolesc Behavior. (2015) 3(2):2–7. 10.4172/2375-4494.1000189

[29] Fisseha HZ Lerebo W Teferi KA. Substance abuse and predictors of risky sexual behavior among students in axum university. J Addict Res Ther. (2015) 6(1):1–6. 10.4172/2155-6105.100020626925299

[30] Lamade K Tesfaye TG Berhanu A W/tsadik M. Sexual and risky sexual behaviors experience of Wolkite University students Central Ethiopia-Descriptive cross-sectional study. Res Sq. (2019). 10.21203/rs.2.12702/v1

[31] Derbie A Assefa M Mekonnen D Biadglegne F. Risky sexual behaviour and associated factors among students of debre-tabor university. Ethipia J Health Dev. (2016) 30(1):11–8. https://www.ajol.info/index.php/ejhd/article/view/147295

[32] Perera UAP Abeysena C. Prevalence and associated factors of risky sexual behaviors among undergraduate students in state universities of Western Province in Sri Lanka: a descriptive cross sectional study. Reprod. Health. (2018) 15(1):105. 10.1186/s12978-018-0546-z 29866189 PMC5987645

[33] Brian AJI Umeononihu O Echendu AD Eke N. Sexual behaviour among students in a tertiary educational institution in Southeast Nigeria. Adv Reprod Sci. (2016) 4(3):87–92. 10.4236/arsci.2016.43010

[34] Mamo K Admasu E Berta MM. Prevalence and associated factors of risky sexual behavior among debremarkos university regular undergraduate students. J Health Med Nurs. (2016) 13:43–6.

[35] Mavhandu-Mudzusi AH tesfay Asgedom T. The prevalence of risky sexual behaviours amongst undergraduate students in Jigjiga university. Health SA Gesondheid. (2016) 4:179–86.

[36] Mulu W Yimer M Abera B. Sexual behaviours and associated factors among students at Bahir Dar University: a cross sectional study. Reprod Health. (2014) 11(1):84. 10.1186/1742-4755-11-84 25481831 PMC4271440

[37] Wordofa Dea. Sexual risk behaviours and associated factors among undergraduate Madawalabu university students epidemiology: open access. JHIA Africa Thesis Bank. (2016) 5(4):3–5. http://thesisbank.jhia.ac.ke/6856/

[38] Adefuye A Abiona TC Balogun JA Amosun SL Frantz J Yakut Y. Perception of risk of HIV and sexual risk behaviors among students in the United States, Turkey and South Africa. J Soc Aspects HIV/AIDS. (2012) 8(1):19–26. 10.1080/17290376.2011.9724980 23237642

[39] Golbasi Z Kelleci M. Sexual experience and risky sexual behaviours of Turkish university students. Arch Gynecol Obstet. (2011) 283(3):531–7. 10.1007/s00404-010-1363-y 20135137

[40] Sales WB Caveião C Visentin A Mocelin D Costa P Simm E. Risky sexual behavior and knowledge of STIs/AIDS among university health students. Rev Enferm Ref. (2016) 4(10):19–28. 10.12707/RIV16019

[41] Delavande A Kohler H-P. HIV/AIDS-related expectations and risky sexual behaviour in Malawi. Rev Econ Stud. (2015) 83(1):118–64. 10.1093/restud/rdv028

[42] Gong E. HIV testing and risky sexual behaviour. Econ J. (2014) 125(582):32–60. 10.1111/ecoj.12125

[43] Woldeyohannes D Asmamaw Y Sisay S Hailesselassie W Birmeta K Tekeste Z. Risky HIV sexual behavior and utilization of voluntary counseling and HIV testing and associated factors among undergraduate students in Addis Ababa, Ethiopia. BMC Public Health. (2017) 17(1):121. 10.1186/s12889-017-4060-y 28122536 PMC5267391

[44] Ajide KB Balogun FM. Knowledge of HIV and intention to engage in risky sexual behaviour and practices among senior school adolescents in Ibadan, Nigeria. Arch Basic Appl Med. (2018) 6(1):3–8. PMID: ; PMCID: 30294661 PMC6169801

[45] Ren Z Zhou Y Liu Y. Factors associated with unsafe sexual behavior among sexually active Chinese university students, Hebei province, 2019. BMC Public Health. (2021) 21(1):1904. 10.1186/s12889-021-11992-2 34670556 PMC8529721

[46] Adriane K. The Effects of HIV/AIDS Knowledge During Adolescence. United States: University of Michigan (2014).

[47] UNAIDS. Youth and HIV Population-Based Surveys From 2011 to 2016. Geneva: United Nations Programme on HIV/AIDS (UNAIDS) (2018).

[48] Singh S Bankole A Woog V. Evaluating the need for sex education in developing countries: sexual behaviour, knowledge of preventing sexually transmitted infections/HIV and unplanned pregnancy. Sex Educ. (2005) 5(4):307–31. 10.1080/14681810500278089

[49] Kaggwa MM Muwanguzi M Najjuka SM Nduhuura E Kajjimu J Mamun MA Risky sexual behaviours among Ugandan university students: a pilot study exploring the role of adverse childhood experiences, substance use history, and family environment. PLoS One. (2022) 17(11):e0277129. 10.1371/journal.pone.0277129 36383509 PMC9668123

[50] Yi S Te V Pengpid S Peltzer K. Social and behavioural factors associated with risky sexual behaviours among university students in nine ASEAN countries: a multi-country cross-sectional study. SAHARA-J: J Soc Aspects HIV/AIDS. (2018) 15(1):71–9. 10.1080/17290376.2018.1503967 PMC6070966 30058474

[51] WHO. Alcohol Use and Sexual Risk Behaviour: A Cross-Cultural Study in Eight Countries. Geneva, Switzerland: World Health Organization (WHO) (2005). p. 59–78.

[52] Henry J, Kaiser Family Foundation. Substance use and risky sexual behavior: attitudes and practices among adolescents and young adults. Am J Health Educ. (2002) 33(5):24–5. 10.1080/19325037.2002.10604750

[53] Tura G Alemseged F Dejene S. Risky sexual behavior and predisposing factors among students of Jimma University, Ethiopia. Ethiop J Health Sci. (2012) 22(3):170–80. PMID: 23209351 PMC3511895

[54] Adimora DE Akaneme IN Aye EN. Peer pressure and home environment as predictors of disruptive and risky sexual behaviours of secondary school adolescents. Afr Health Sci. (2018) 18(2):218–26. 10.4314/ahs.v18i2.4 30602946 PMC6306969

[55] Ndagijimana E Biracyaza E Nzayirambaho M. Risky sexual behaviors and their associated factors within high school students from collège saint André in Kigali, Rwanda: an institution-based cross-sectional study. Front Reprod Health. (2023) 5:1029465. 10.3389/frph.2023.1029465 36936133 PMC10020213

[56] Wana GW Arulogun O Roberts A Kebede AS. Predictors of risky sexual behaviour among pre-college students in Adama town, Ethiopia. Pan Afr Med J. (2019) 33:135. 10.11604/pamj.2019.33.135.18068 31558934 PMC6754848

[57] Fekadu Wakasa B Oljira L Demena M Demissie Regassa L Binu Daga W. Risky sexual behavior and associated factors among sexually experienced secondary school students in Guduru, Ethiopia. Prev Med Rep. (2021) 23:159–64. 10.1016/j.pmedr.2021.101398 PMC8142265 34040934

[58] Cherie A Berhane Y. Peer pressure is the prime driver of risky sexual behaviors among school adolescents in Addis Ababa, Ethiopia. World J AIDS. (2012) 2(03):159–64. 10.4236/wja.2012.23021

[59] Harkness EL Mullan B Blaszczynski A. Association between pornography use and sexual risk behaviors in adult consumers: a systematic review. Cyberpsychol Behav Soc Netw. (2015) 18(2):59–71. 10.1089/cyber.2014.0343 25587721

[60] Afriyie J Essilfie ME. Association between risky sexual behaviour and HIV risk perception among in-school adolescents in a municipality in Ghana. Ghana Med J. (2019) 53(1):29–36. 10.4314/gmj.v53i1.5 31138941 PMC6527831

[61] Nkwonta CA Harrison SE. HIV knowledge, risk perception, and testing behaviors among college students in South Carolina. J Am Coll Health. (2023) 71(1):274–81. 10.1080/07448481.2021.1891078 33759714

[62] Njabulo N Pranitha M. Perception of risk of HIV infections and sexual behavior of the sexually active university students in Zimbabwe. J Soc Aspects HIV/AIDS. (2014) 11(1):42–50. 10.1080/17290376.2014.886082 PMC4272097 24921968

[63] CDC. Youth Risk Behavior Survey. Atlanta: CDC (2018). p. 8–10.

[64] Shiferaw Y Alemu A Assefa A Tesfaye B Gibermedhin E Amare M. Perception of risk of HIV and sexual risk behaviors among university students: implication for planning interventions. BMC Res Notes. (2014) 7(1):162. 10.1186/1756-0500-7-162 24642193 PMC3974211

[65] Stutts LA Robinson PA Witt B Terrell DF. Lost in translation: college students’ knowledge of HIV and PrEP in relation to their sexual health behaviors. J Am Coll Health. (2022) 70(2):561–7. 10.1080/07448481.2020.1757679 32407199

[66] Morales A Espada JP Orgilés M Escribano S Johnson BT Lightfoot M. Interventions to reduce risk for sexually transmitted infections in adolescents: a meta-analysis of trials, 2008–2016. PLoS One. (2018) 13(6):e0199421. 10.1371/journal.pone.0199421 29953546 PMC6023153

[67] Michielsen K Beauclair R Delva W Roelens K Van Rossem R Temmerman M. Effectiveness of a peer-led HIV prevention intervention in secondary schools in Rwanda: results from a non-randomized controlled trial. BMC Public Health. (2012) 12(1):729. 10.1186/1471-2458-12-729 22938717 PMC3504526

[68] Sani AS Abraham C Denford S Ball S. School-based sexual health education interventions to prevent STI/HIV in sub-Saharan Africa: a systematic review and meta-analysis. BMC Public Health. (2016) 16:1–26. 10.1186/s12889-016-3715-4 27724886 PMC5057258

[69] Rohrbach LA Berglas NF Jerman P Angulo-Olaiz F Chou C-P Constantine NA. A rights-based sexuality education curriculum for adolescents: 1-year outcomes from a cluster-randomized trial. J Adolesc Health. (2015) 57(4):399–406. 10.1016/j.jadohealth.2015.07.004 26403840

[70] Millanzi WC Osaki KM Kibusi SM. The effect of educational intervention on shaping safe sexual behavior based on problem-based pedagogy in the field of sex education and reproductive health: clinical trial among adolescents in Tanzania. Health Psychol Behav Med. (2022) 10(1):262–90. 10.1080/21642850.2022.2046474 35251774 PMC8896187

[71] Boti Sidamo N Hussen S Shegaze Shimbre M Zerihun E Godana Boynito W Abebe S Effectiveness of curriculum-based sexual and reproductive health education on healthy sexual behaviors among year one students at Arba Minch University: a quasi-experimental study. PLoS One. (2023) 18(10):e0288582. 10.1371/journal.pone.0288582 37906542 PMC10617698

[72] Pfeiffer C Kleeb M Mbelwa A Ahorlu C. The use of social media among adolescents in Dar es Salaam and Mtwara, Tanzania. Reprod Health Matters. (2014) 22(43):178–86. 10.1016/S0968-8080(14)43756-X 24908469

[73] Doubova SV Martinez-Vega IP Infante-Castañeda C Pérez-Cuevas R. Effects of an internet-based educational intervention to prevent high-risk sexual behavior in Mexican adolescents. Health Educ Res. (2017) 32(6):487–98. 10.1093/her/cyx074 29177452

[74] Licata F Angelillo S Nobile CGA Di Gennaro G Bianco A. Understanding individual barriers to HIV testing among undergraduate university students: results from a cross-sectional study in Italy. Front Med. (2022) 9:882125. 10.3389/fmed.2022.882125 PMC9063657 35514754

[75] Cassidy C Steenbeek A Langille D Martin-Misener R Curran J. Designing an intervention to improve sexual health service use among university undergraduate students: a mixed methods study guided by the behaviour change wheel. BMC Public Health. (2019) 19(1):1734. 10.1186/s12889-019-8059-4 31878901 PMC6933635

[76] Heard E Auvaa L Pickering C. Love bugs: promoting sexual health among young people in Samoa. Health Promot J Austr. (2015) 26(1):30–2. 10.1071/HE14055 25436987

[77] Licata F Angelillo S Oliverio A Di Gennaro G Bianco A. How to safeguard university students against HIV transmission? Results of a cross-sectional study in southern Italy. Front Med. (2022) 9:903596. 10.3389/fmed.2022.903596 PMC9263725 35814762

[78] Johnson M Afonina L Haanyama O. The challenges of testing for HIV in women: experience from the UK and other European countries. Antiviral Ther. (2013) 18(Suppl 2):19–25. 10.3851/IMP2637 23784671

[79] Mason-Jones AJ Mathews C Flisher AJ. Can peer education make a difference? Evaluation of a South African adolescent peer education program to promote sexual and reproductive health. AIDS Behav. (2011) 15(8):1605–11. 10.1007/s10461-011-0012-1 21809049

